# Commentary on: “Number-space mapping in the newborn chick resembles humans' mental number line”

**DOI:** 10.3389/fpsyg.2015.00352

**Published:** 2015-03-27

**Authors:** Caroline B. Drucker, Elizabeth M. Brannon

**Affiliations:** ^1^Center for Cognitive Neuroscience, Duke UniversityDurham, NC, USA; ^2^Department of Neurobiology, Duke UniversityDurham, NC, USA; ^3^Department of Psychology and Neuroscience, Duke UniversityDurham, NC, USA

**Keywords:** numerical cognition, spatial cognition, mental number line, birds, culture

When you think about the numbers one through twenty, what do you see? Most people visualize numbers laid out in space from left to right. In other words, we have a “mental number line” (Restle, 1970; Seron et al., 1992). People are actually faster at making judgments about small numbers when they are presented on the left and large numbers on the right, providing strong evidence that number is mentally mapped onto space in adults (Dehaene et al., 1993). This oft-replicated psychophysical phenomenon (Fias and Fischer, 2005) is reduced or even reversed in cultures that read from right to left, suggesting that the mental number line is a byproduct of culturally specific experiences (Zebian, 2005; Shaki and Fischer, 2008; Shaki et al., 2009). An alternative possibility is that even though the mental number line can be influenced by cultural practices it has a more fundamental place in the mind.

Recent evidence that preverbal infants (de Hevia and Spelke, 2010), birds (Rugani et al., 2007, 2010, 2011), chimpanzees (Adachi, 2014), and monkeys (Drucker and Brannon, 2014) map number onto space endorses a biological basis for the mental number line. However, these developmental and comparative studies omit a key feature of the mental number line in humans: a given number is “large” in some contexts and “small” in others. In an elegant new experimental study, Rugani and colleagues demonstrate that newborn domestic chicks (*Gallus gallus*) also show context-dependent spatial-numerical mapping (Rugani et al., 2015).

Rugani and colleagues first trained 3-day-old chicks to walk around a panel for a reward (Figure 1). To establish a numerical context, the panel always displayed the same number of items for a given chick: either five or twenty. Later that day, chicks were placed in an arena with two panels in front of them to the left and right displaying the same number of items. If the chick had been trained with five items, both panels either had two or eight items; if it had been trained with twenty items, the panels both showed eight or thirty-two items. A reward was placed behind each of the two identical panels. If chicks did not mentally map number onto space they should have had no preference. Astonishingly, when the number displayed on the panels was lower than the training number, chicks tended to walk to the left panel; when it was higher than the training number, they walked to the right. Crucially, the side they preferred when the test number was eight depended on training context: chicks trained with five walked to the right, whereas chicks trained with twenty walked to the left.

**Figure 1:**
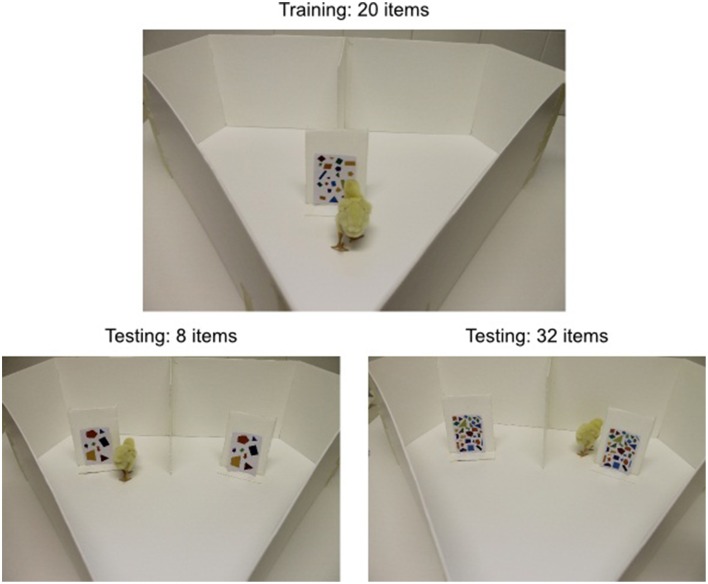
**A chick performing the task developed by Rugani et al. (2015)**. Training with a single 20-item panel is shown at the top. The two different testing conditions are shown at the bottom, with the low-number test (8 vs. 8) shown at left and the high-number test (32 vs. 32) shown at right. The images depict a control condition where shape, size, and color of the items were varied. Photographs courtesy of Rosa Rugani.

Control experiments ruled out other non-numerical features of the visual displays that might have influenced the chicks' choices. First Rugani and colleagues randomized the shape, color, and size of the items on the panels. Next they equated the total surface area as well as the overall spatial extent occupied by the items on each training and testing panel. Finally they equated the total perimeter and density of the items on each training and testing panel. Through all of these conditions, chicks maintained their preference for the left (or right) panel when test numbers were smaller (or larger, respectively) than the trained value. These results suggest that chicks map number—rather than some other continuous parameter that co-varies with number—onto space. In adult humans, a host of continua are mapped onto space, including temporal duration (Vallesi et al., 2008), volume of liquid (Kirjakovski and Utsuki, 2012), and even degree of emotional expression (Holmes and Lourenco, 2011). Such findings suggest a more generalized magnitude-space mapping, or a mental magnitude line. Future studies should explore whether other animals map any type of magnitude besides number to space.

Rugani and colleagues' findings provide clear evidence that chicks map number to space, and that as in humans, this mapping is context-dependent. Moreover, the 3-day-old chicks had little experience with numerical stimuli, had no exposure to cultural artifacts like keyboards portraying numbers in a line, and definitely did not read written text—largely ruling out the possibility that number-space mappings are learned. Rather, the tendency to map number to space seems to be an intrinsic part of representing number or more generally magnitude. Why might animals map number or magnitudes onto space?

The explanation provided by Rugani and colleagues involves hemispheric asymmetries in bird brain function. They propose that right hemisphere dominance for numerical processing produces increased right hemisphere activation—and thus increased leftward attention—when considering numerical information, which could cause birds to begin enumerating from the left (see also Vallortigara, 2012). Another possibility is that numerical representations are spatially organized in the brain. Recent functional neuroimaging evidence suggests that there is a topographical arrangement of numerical magnitudes in human parietal cortex (Harvey et al., 2013). However, this cortical map has only been found in humans, and only for the numbers one to seven.

Another idea is that numerical and spatial cognition rely on common neural circuits (Hubbard et al., 2005). The same regions of parietal cortex—in particular, the lateral and ventral intraparietal areas—play a role in processing both number and spatial attention in primates. Activation of these regions by numerical information could therefore lead to shifts in spatial attention. This hypothesis is attractive from an evolutionary perspective, since navigating space is a fundamental problem faced by all animals. Rather than developing new cognitive systems to deal with abstract concepts of “less” and “more,” it would be more efficient to re-use the system already in place for processing space (Holmes and Lourenco, 2011). Considering numerical processing as an exaptation (Gould and Vrba, 1982) of spatial processing could explain the functional link between these two systems.

An important caveat to these hypotheses is that one-to-one homologies between avian and mammalian brain regions remain elusive (Jarvis et al., 2005). In particular, number representation has not been localized in the avian brain. Exploring where and how bird brains process numbers could shed light on the origins of the mental number line.

## Conflict of interest statement

The authors declare that the research was conducted in the absence of any commercial or financial relationships that could be construed as a potential conflict of interest.
